# Intracranial closed-loop neuromodulation as an intervention for neuropsychiatric disorders: an overview

**DOI:** 10.3389/fpsyt.2025.1479240

**Published:** 2025-01-30

**Authors:** Jenna Langbein, Ujwal Boddeti, Weizhen Xie, Alexander Ksendzovsky

**Affiliations:** ^1^ Department of Neurosurgery, School of Medicine, University of Maryland, Baltimore, MD, United States; ^2^ Department of Psychology, University of Maryland, College Park, MD, United States

**Keywords:** closed loop neuromodulation, neuropsychaitric disorders, neural circuit, neural network, DBS (deep brain stimulation), RNS = responsive neurostimulation

## Abstract

Recent technological advances in intracranial brain stimulation have enhanced the potential of neuromodulation for addressing neuropsychiatric disorders. We present a review of the methodology and the preliminary outcomes of the pioneering studies exploring intracranial biomarker detection and closed-loop neuromodulation to modulate high-symptom severity states in neuropsychiatric disorders. We searched PubMed, Scopus, Web of Science, Embase, and PsycINFO/PsycNet, followed by the reference and citation lists of retrieved articles. This search strategy yielded a total of 583 articles, of which 5 articles met the inclusion criteria, focusing on depression, obsessive-compulsive disorder, post-traumatic stress disorder, and binge eating disorder. We discuss the methodology of biomarker identification, the biomarkers identified, and the preliminary treatment outcomes for closed-loop neuromodulation. Successful biomarker identification hinges on investigating across various setting. Targeted neuromodulation, either directed at the biomarker or within its associated neural network, offers a promising treatment approach. Future research should seek to understand the mechanisms underlying the effects of neuromodulation as well as the long-term viability of these treatment effects across different neuropsychiatric conditions.

## Introduction

Neuropsychiatric disorders have a profound impact on the mental health of approximately 970 million individuals, resulting in healthcare costs reaching into the trillions (1). Despite the availability of therapeutic and pharmaceutical treatments, only a fraction of patients respond positively to these conventional interventions. Standard of care achieves remission in fewer than half of patients with obsessive-compulsive disorder (OCD) (2), and a mere 30% of individuals grappling with depression attain remission (3, 4). These challenges are further compounded by issues such as adverse drug effects (5), logistical complexities in administering treatments—especially with psychotherapeutic options—and suboptimal treatment adherence (6). While symptoms are remarkably heterogeneous, treatments are often delivered based on the diagnosis, rather than the presenting functional symptoms. As neuropsychiatric diseases are increasingly understood to be disorders of dysfunctional neural circuits, there arises a need to understand the patient’s unique circuit-level pathophysiology, particularly for treatment-resistant individuals (7).

Among various alternatives, neural circuitry modulation through techniques like transcranial electrical or magnetic stimulation (tES/TMS) has emerged as a promising treatment for neuropsychiatric disorders (8–12). These interventions often target neural network dysfunction associated with neuropsychiatric conditions, potentially alleviating corresponding symptoms, such as in depression and OCD (13). However, transcranial approaches encounter inherent constraints. For example, the application of transcranial techniques restricts access to deep neural structures intricately associated with neuropsychiatric disorders, including the amygdala-hippocampus complex and nucleus accumbens (14). Further technical challenges, such as the complexity of simultaneously recording electroencephalography (EEG) and administering tES/TMS, present obstacles. Although office-based administration is effective (11), this setting is not always the most conducive, given the inaccessibility for some patients, the temporal variation in symptoms, and the inadequacy of accommodating diverse environments. This notably impacts patients whose treatment necessitates addressing specific exposures and contextual factors, such as individuals suffering from OCD and addiction.

To mitigate these issues, recent research has explored two novel approaches. First, to better target relevant neuropsychiatric structures, recent studies have attempted to obtain direct electrophysiology using implanted intracranial electrodes (15–21). Intracranial EEG (iEEG) captures neural circuits in high spatiotemporal detail (22), available both on the brain’s surface with subdural electrodes or directly into brain structures using depth electrodes (23). This approach is commonly used as a part of surgical epilepsy evaluation in those with drug-resistant epilepsy and has more recently been applied to understanding the neural correlates of neuropsychiatric diseases, such as depression (24), OCD (25), and psychosis (26).

Second, to translate these in-lab findings into more flexible at-home treatments, recent research has further explored chronically implanted closed-loop neurostimulation devices such as Responsive Neurostimulation (RNS) or adaptive deep brain stimulation (DBS) to achieve closed-loop stimulation. As opposed to an open-loop system where stimulation is delivered at a predetermined interval irrespective of neurophysiological state, closed-loop stimulation involves the detection of biomarkers related to symptom severity and the subsequent delivery of time-specific stimulation in response, within milliseconds (27). Responsive neurostimulation refers to the delivery of stimulation for a fixed duration after a triggering event, while adaptive neurostimulation involves adjustment of therapeutic parameters based upon changes to the neural signals (28). These options make it possible for patients to engage in regular life activities in an at-home setting while receiving treatment benefits, as well as paralleling treatment to the natural variations in symptom severity.

Despite great potential, many unknowns persist regarding the mechanisms, efficacy, and long-term effects of these intracranial neuromodulation effects in neuropsychiatric conditions. Therefore, to systematically map the existing literature and to better understand treatment opportunities for researchers and clinicians, we conducted a review of recent clinical studies employing intracranial, closed-loop neurostimulation as a novel treatment for neuropsychiatric diseases. Our objectives include: (1) reporting and summarizing the approaches to biomarker discovery; (2) reporting and summarizing the neurostimulation protocols and preliminary treatment outcomes; and (3) providing recommendations for consideration for future studies.

## Materials and methods

### Literature search

**Figure 1:**
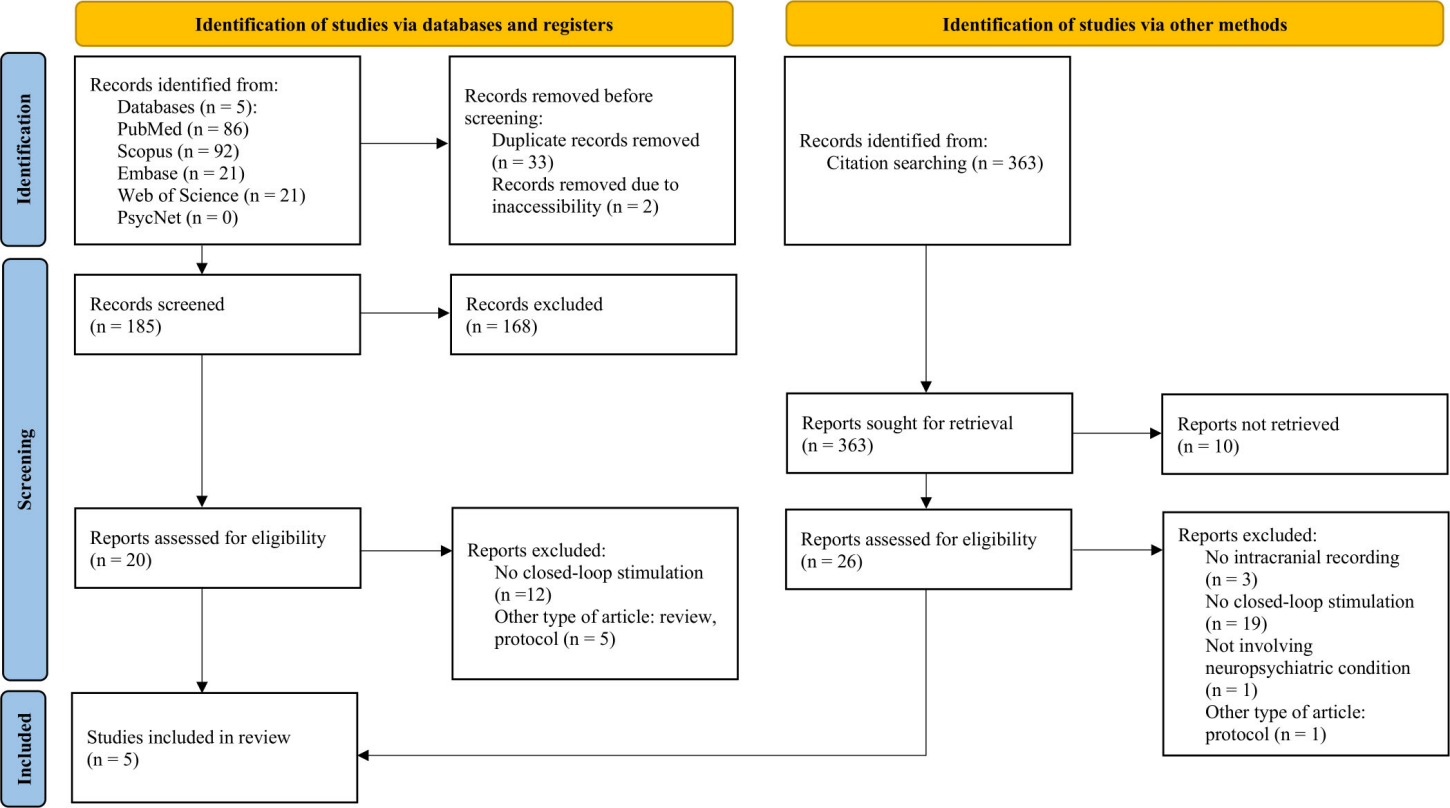
Flow diagram (30) for systematic literature search and selection of articles, created with PRISMA 2020 Shiny App Online Tool (56).

A preliminary search of MEDLINE, the Cochrane Database of Systematic Reviews, and JBI Evidence Synthesis found no existing reviews on the topic. This review draws inspiration from the Joanna Briggs Institute methodology (29), and the Preferred Reporting Items for Systematic Reviews and Meta-Analyses extension for Scoping reviews (PRISMA-ScR) guidelines (30). We used a two-stage search strategy to identify relevant, published articles that identify intracranial electrophysiological biomarkers for closed-loop neuromodulation of neuropsychiatric diseases. In the first stage, we searched the following databases: PubMed, Scopus, Web of Science, Embase, and PsycInfo/PsycNet. Search terms included keywords such as “intracranial recording,” “closed-loop neuromodulation,” and “neuropsychiatric disorders” (see **Supplementary Table 1** for details). This yielded a total of 185 articles, of which three articles were eligible for the review. In the second stage, we used a forward and backward snowballing approach to optimize our search, which involved searching reference and citation lists of retrieved articles for additional relevant studies (31). As of February 2024, this two-stage search strategy yielded a total of 583 articles which were reviewed for relevance and eligibility based on inclusion and exclusion criteria (**Figure 1**).

### Criteria for inclusion and exclusion

Articles were included if they (1) included iEEG recordings, whether from a chronically implanted device or through sub-chronic iEEG placement; (2) correlated these iEEG features or biomarkers with at least one neuropsychiatric disorder symptom; and (3) applied electrical stimulation to affect the severity of the identified neuropsychiatric disorder symptom through closed-loop neuromodulation. Articles were excluded if they (1) did not feature an intracranial biomarker that was associated with a symptomatic state; (2) did not perform closed-loop intracranial stimulation in response to that biomarker; (3) were animal studies, nonclinical or technical articles, review articles, editorials, or conference presentation abstracts; or (4) were not written in English. Each article excluded was coded according to the numbers above (ex. - 1 if there was no mention of an intracranial biomarker). Irrelevant articles were excluded at two levels: (1) by reviewing the title and abstract, or (2) after full review (see **Figure 1** for details).

### Extraction of study characteristics

Key characteristics of the included studies were recorded following an *a priori* coding scheme, including (1) background information on the articles, such as title, author, publication year, and number of patients; (2) electrode information, such as electrode recording device and location of implanted electrodes; (3) biomarker identification and selection methods; (4) symptom-specific biomarker findings; and (5) caveats. For results related to the stimulation therapy, key characteristics were: (1) target; (2) stimulation protocol, including the device used and stimulation parameters; and (3) treatment outcomes, including primary endpoint and remission criteria, and whether both were met. All the *a priori* criteria were coded as columns in a data-charting form and were filled out by two separate reviewers. Results were summarized in tabular format (see **Tables 1**–**4**).

**Table 1:** Study characteristics.

Study	Authors and year	Study type and number of patients	Patient characteristics
Closed-loop neuromodulation in an individual with treatment-resistant depression	Scangos et al., 2021 (33)	Case report, n=1	36-year-old female with treatment-resistant depression
Long-term ecological assessment of intracranial electrophysiology synchronized to behavioral markers in obsessive-compulsive disorder	Provenza et al., 2021 (32)	Case series, n=5 patients with OCD, n=3 for patients with intracranial recordings via Summit RC+S	31 to 40 years old 3 females, and 2 males with treatment-resistant OCD; multiple comorbidities each (PTSD, Tourette syndrome, depression, and bipolar II)
Responsive deep brain stimulation guided by ventral striatal electrophysiology of obsession durably ameliorates compulsion	Nho et al., 2024 (36)	Case report, n=1	31-year-old female with OCD and comorbid epilepsy
A pilot study of closed-loop neuromodulation for treatment-resistant post-traumatic stress disorder	Gill et al., 2023 (34)	Case series, n=8 total, n=2 with treatment-resistant PTSD	Average age 38 years old, both males with treatment-resistant PTSD
Pilot study of responsive nucleus accumbens deep brain stimulation for loss-of-control eating	Shivacharan et al., 2022 (35)	Case series, n=2	45 and 46 years old, both females with treatment-refractory binge eating disorder
Identification of a personalized intracranial biomarker of depression and response to DBS therapy	Frank et al., 2021 (40)	Case report, n=1	51-year-old male with treatment-refractory OCD and depression

OCD, obsessive-compulsive disorder; PTSD, post-traumatic stress disorder; DBS, deep brain stimulation.

**Table 2:** Summary of main pertinent findings.

Authors and year; disease	Electrode recording method	Implanted electrodes	Symptom provocation	Methodology of biomarker selection, hypothesis-driven and/or data-driven	Main biomarker findings	Limitations
Scangos et al., 2021 (33) Depression	sEEG and RNS	bilateral orbitofrontal cortex, amygdala*, hippocampus, ventral capsule/ventral striatum*, and subgenual cingulate cortex *indicates location of electrodes from RNS implant after initial sEEG	- naturalistic: during hospitalization with sEEG, recordings were obtained during a variety of activities (“recalling life events, watching movies, and using social media”) to replicate natural variations in mood - naturalistic: with RNS implanted, participant performed at-home surveys to record symptom state time-locked to recording as well as in the laboratory setting	feature selection was based on ANOVA F values, and models were built using penalized logistic regression, trained on 80% of the dataset, and tested on the remaining 20% data driven	- bilateral amygdala gamma power via sEEG was sufficient to detect high symptom severity state, evaluated with two cross-validated machine learning models (accuracy: mean 0.77, sd = 0.09; area under curve mean = 0.82, sd = 0.11) - RNS detections of amygdala gamma power were found to be 87% predictive of symptom severity state and highly correlated with VAS-D (r2 = 0.59, P = 1.2 × 10−4), VAS-A (r2 = 0.52, P = 4.6 × −4) and HAMD-6 (r2 = 0.65, P = 1.98 × 10−5)	- clinicians were not always blinded to stimulation location and parameters, which could have affected therapeutic response - participant could have detected non-affective sensations experienced during stimulation, which may have influenced the participant’s response to treatment - participant self-selected for study, which may represent selection bias
Provenza et al., 2021 (32) OCD	DBS	ventral striatum or bed nucleus of the stria terminalis	- naturalistic: participants reported OCD symptom severity at home, with time synchronization to intracranial recordings - in lab: participated in exposure-response therapy sessions which aimed to provoke OCD-related stress	average normalized spectral power in predefined frequency bands was fit to correlate with OCD symptom intensity hypothesis and data driven	delta band power showed a strong negative correlation with OCD symptom intensity in the bilateral VC/VS (right, r=-0.593, left, r=-0.557) in participant 4	- bipolar contact pairs sensing neural activity from white vs gray matter depending on placement, which could impact the interpretation of recordings - self-reported symptoms are not always reliable or can exhibit reporting bias as participants tend to comply with recordings when symptoms are less severe and not as much when symptomatic - only includes some of the participants in an analysis of the longitudinal data to extract a particular biomarker; other participants showed variable correlations between spectral power and exposure provocation-related distress ratings
Nho et al., 2024 (36) OCD	RNS	ventral striatum	- naturalistic: self-triggered storage of recordings during a state of obsessive thoughts - in lab: provoked distress through exposure to triggering objects and through a virtual reality task	correlated obsessive state with specific frequencies contributing to the peaks observed in the area under the curve analysis hypothesis and data driven	low frequency signal, <15 Hz, corresponded with obsessive state in both ambulatory and provocatory settings	- broader range in frequencies in ambulatory data as compared to in-lab testing - difficulty deciphering between signals corresponding to obsessive and compulsive-related behaviors - limitations of device storage, necessitating balance between recording seizure-related and OCD-related data

sEEG, stereotactic encephalography depth electrodes; RNS, responsive neurostimulation system; VAS-D, visual analog scale depression; VAS-A, visual analog scale anxiety; HAMD-6, Hamilton Depression Rating Scale-6; OCD, obsessive-compulsive disorder; VC/VS, ventral capsule/ventral striatum.

**Table 3:** Summary of main pertinent findings.

Authors and year; disease	Electrodes recording method	Implanted electrodes	Symptom provocation	Methodology of biomarker selection, hypothesis-driven and/or data-driven	Main biomarker findings	Limitations
Gill et al., 2023 (34) PTSD	RNS	bilateral amygdala	- naturalistic: self-reported symptom exacerbations - in lab: emotional image and emotional narrative tasks intended to provoke symptoms	linear mixed effects and cluster permutation to differentiate frequency power differences in particular emotional states hypothesis and data driven	increases in amygdala theta (5-9 Hz) band power corresponded with negative emotional image viewing, listening to recorded narratives of traumatic experiences, and during natural symptom exacerbations	- differential treatment patterns based on individual biomarkers limit inter-participant analysis
Shivacharan et al., 2022 (35) Binge eating	DBS	nucleus accumbens	- naturalistic: participants self-triggered storage of recordings when they had a craving and were about to eat - in lab: multi-item buffet to model environment designed to trigger an episode of loss of control eating	used ANOVA to show differences in band powers during different hunger states hypothesis and data driven	low-frequency, 2-8 Hz, delta band power increases present in the nucleus accumbens immediately preceding an episode of LOC eating	- within-subject control, comparing LOC eating episodes to eating without significant craving, could benefit from control with individuals who do not have binge eating - biomarker was found to have a high sensitivity, but lower specificity (overlapped with normal physiologic processes such as sleep)
Frank et al., 2021 (40) OCD and depression	DBS	anterior limb of the internal capsule and bed nucleus of stria terminalis	- naturalistic: self-triggered storage with experience of symptom exacerbation - in lab: cycled stimulation off and on accompanying LFP recordings	spearman correlations between power spectral density across frequency bands and VAS scores, validated through permutation tests, bootstrap analysis, and cross-validation hypothesis and data driven	low gamma power (25-50 Hz) and high gamma power (50-100 Hz) in BNST inversely correlated with depression severity	- by nature as a Letter to the Editor, this article represents preliminary communication of findings, rather than comprehensive analysis, with limited methodological details - lack of longitudinal data - reports solely on depression but monitored for symptoms of OCD as well

RNS, responsive neurostimulation system; PTSD, post-traumatic stress disorder; DBS, deep brain stimulation; LOC, loss of control; OCD, obsessive compulsive disorder.

**Table 4:** Effects of treatment with neuromodulation.

Study	Target	Device	Stimulation parameters	Method of tracking treatment efficacy*	Primary treatment endpoint	Primary endpoint met?	Remission criteria	Remission criteria met?
Closed-loop neuromodulation in an individual with treatment-resistant depression	right VC/VS	RNS	100 Hz 120 µs pulse width 1mA 6s interval duration	clinical assessment - HAMD-6 and VAS-D (and periodic MADRS- clinician interview) research assessment - change in amygdala gamma power post-stimulation	Change in MADRS score	Yes	MADRS score <10	100% (n=1)
Long-term ecological assessment of intracranial electrophysiology synchronized to behavioral markers in obsessive-compulsive disorder	VS or BNST	Activa PC+S	150 Hz 5.0 - 5.5 V 120 µs pulse width	physiologic - recorded face to estimate positive affect (AFAR detection) and head velocity, as objective measures of anxiolytic and anxiogenic responses; other physiologic measures like EKG, blood volume pulse, and EEG clinical - Y-BOSC and self-report intensity	Change in Y-BOCS score of 35% reduction from baseline	Yes	Y-BOCS score <= 12	40% (n=5)
		Summit RC+S	150.6 Hz 5.0 - 6.0 mA 120 - 210 µs pulse width					
Responsive deep brain stimulation guided by ventral striatal electrophysiology of obsession durably ameliorates compulsion	ventral striatum	RNS	125 Hz 7.0 mA 1000 ms burst duration 7.1 charge density µC/cm2 80 µs pulse width per phase	clinical - self report, Y-BOSC	unspecified	–	–	–
A pilot study of closed-loop neuromodulation for treatment-resistant post-traumatic stress disorder	amygdala	RNS	200 Hz 100 ms 1.0-3.0 mA pulse width 160 µs delivered bilaterally	clinical - CAPS-5 and PCL-5 score research - changes in amygdala theta power post-stimulation	difference of mean CAPS-5 scores; indicative of reliable change when difference scores were greater than or equal to the respective threshold 13 for identifying clinically meaningful change for male combat veterans, defined by Marx et al	Yes	–	–
Pilot study of responsive nucleus accumbens deep brain stimulation for loss-of-control eating	bilateral NAc	RNS	125 Hz Two 5 sec bursts Charge density 0.5 → 1.5 µC/cm2	clinical - self-report frequency of LOC eating events, LOC severity (assessed by ELOCS scale), BED severity physiologic - objective measures of body weight and BMI	At least 50% of subjects exhibited a decrease in the number of LOC eating events per week, assessed via EMA	yes	Fewer than average of four binge eating events per month over the prior consecutive 3 months	50% (n=2)

VC/VS, ventral capsule/ventral striatum; RNS, responsive neurostimulation system; HAMD-6, Hamilton Depression Rating Scale; VAS-D, visual analog scale depression; MADRS, Montgomery-Asberg Depression Rating; BNST, bed nucleus of stria terminalis; AFAR, Automated Facial Affect Recognition; EKG, electrocardiogram; EEG, electroencephalogram; Y-BOSC, Yale-Brown Obsessive-Compulsive Scale; CAPS-5, Clinician-Administered PTSD Scale for DSM-5; PCL-5, PTSD Checklist for DSM-5; NAc, nucleus accumbens; EMA, ecological momentary assessment.

*clinical assessment refers to the clinical scores or the clinician assessment of response to treatment; research assessment refers to the change in the biomarker that was identified within the study as a response to the treatment; physiologic assessment refers to the physiologic changes in response to treatment.

## Results

**Figure 2:**
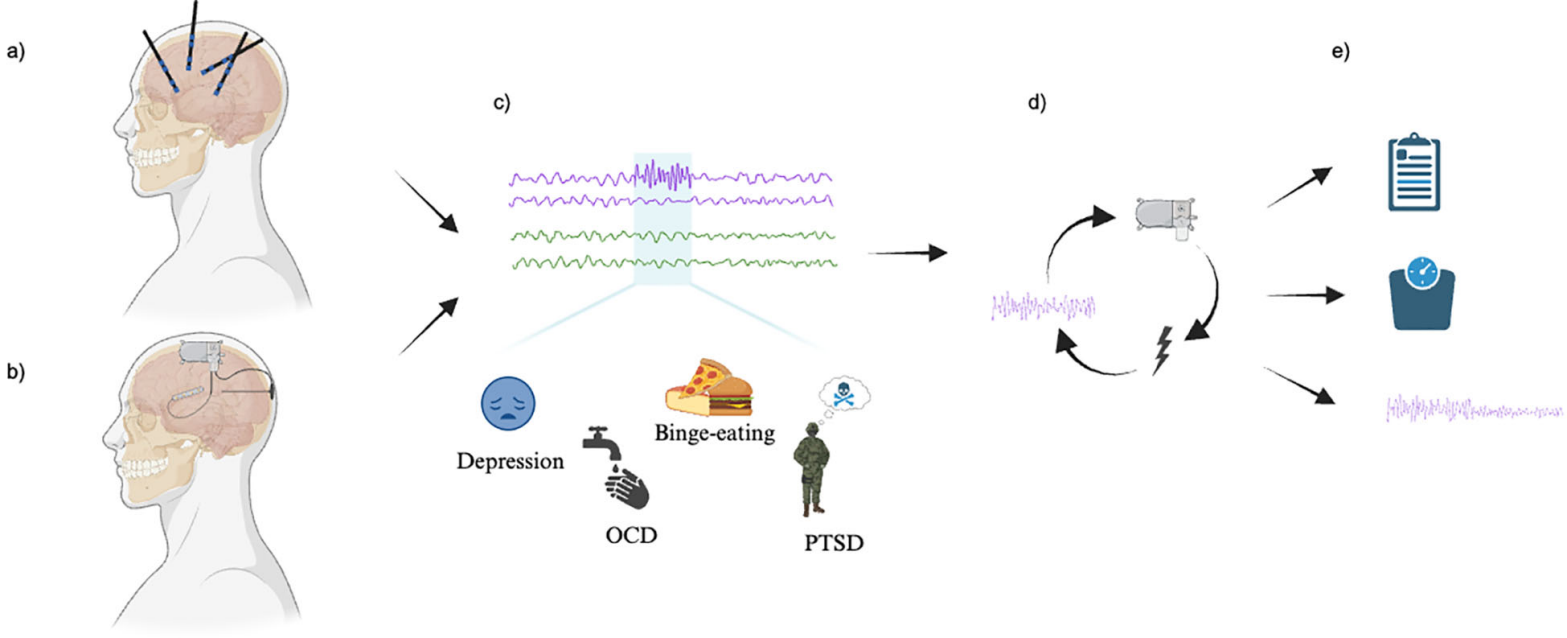
Study paradigm. **(A)** Exploratory mapping versus **(B)** Anatomically targeted recording and stimulation (via closed-loop neuromodulation device electrodes). **(C)** Detection of electrophysiological biomarkers (above) corresponding to a state of high symptom severity. **(D)** Closed-loop neuromodulation as informed by the detected biomarker. **(E)** Change in symptoms, as defined by (top to bottom): clinical measures, physiological measures, and research-defined biomarker changes. Image created with BioRender.com.

Our search yielded a total of 583 articles. After review for duplicates and relevancy, 46 articles were identified, of which 5 were included in this review. The majority of the studies excluded at full review did not apply closed-loop neurostimulation. The included studies, published between 2021 and 2024, had sample sizes ranging from a single case report to 5 participants, all with treatment-resistant neuropsychiatric diseases. Many of the patients had attendant neuropsychiatric and neurological comorbidities. Two articles investigated OCD, one article focused on depression, one article studied PTSD, and another studied binge eating disorder. Each of the studies followed a similar paradigm of intracranial electrophysiological biomarker detection by recording during various symptomatic states and through different settings, including home recordings with self-report symptoms to provide ecological validity, through provocatory behavioral tasks, or therapy sessions (depicted in **Figure 2**). For instance, Provenza and colleagues obtained recordings from self-report logging during symptomatic states, during exposure and response therapy sessions, as well as during behavioral tasks aimed at provoking an obsessive state (32). Details from each study are shared in **Tables 2**, **3**. One study took an additional step to contextualize the biomarker and response to stimulation within the underlying structural and functional connectivity of the subnetwork involved (33).

### Detection of iEEG biomarkers associated with neuropsychiatric disorders

Successful application of intracranial stimulation requires the identification of a reliable biomarker that tracks neuropsychiatric symptoms. This involves two different approaches: exploratory mapping and targeted recording (**Figures 2A, B**). Exploratory mapping involves a wider distribution of implanted electrodes, aiming to identify an implicated region or circuit by linking their electrophysiological features with the severity of neuropsychiatric symptoms through a process akin to conventional seizure foci mapping. Targeted recording, in contrast, involves direct recording from an *a priori* structure based on an assumed or previously tested association between a given anatomical target structure and neuropsychiatric symptoms. Both exploratory mapping and targeted recording approaches in these studies rely on empirical, or data driven, methods to identify biomarkers as therapeutic targets. Each study identified time periods corresponding to symptomatic states and examined electrophysiologic correlates during these phases. While two studies corroborated findings with animal models (34, 35), all studies utilized power spectral analyses to empirically identify relevant biomarkers.

Biomarker selection from either approach involves identifying spectral bands showing power differences that discriminate between high and low symptom severity states (see **Tables 2**, **3** for the methodology of biomarker selection) (28). Four studies correlated neural features to symptom ratings, one of which took advantage of the detection algorithms implicit to the RNS device (36); these algorithms include line length, area, and bandpass detection tools (37). Effectively, these algorithms identify changes in amplitude, frequency, signal energy, rhythmic spiking activity, or some combination of the above, to differentiate the pattern of pathologic activity from normal functioning (37). One study used a more advanced biomarker selection paradigm; this involves dimensionality reduction and predictive models or classifiers to identify relevant neural features (28, 33).

#### Exploratory mapping

Given the lack of studies investigating the electrophysiological correlates of neuropsychiatric disorders, personalized circuitry, and the relative complexity of presenting symptoms, it is difficult to know the best structural targets to detect biomarkers from and/or apply with neuromodulation. Exploratory mapping mitigates these challenges by searching electrophysiological features in a wide range of brain structures to link these features with symptom severity. For example, Scangos and colleagues initially mapped out several cortical and subcortical structures within the depression-associated corticolimbic circuit (38), using depth iEEG electrode probes (also known as stereotactic encephalography, sEEGs) in the bilateral orbitofrontal cortex (OFC), amygdala, hippocampus, ventral capsule/ventral striatum (VC/VS) and subgenual cingulate cortex (SGC) (33). For their pilot patient, they found that bilateral amygdala gamma power was sufficient to detect the high symptom severity state (33). Subsequent recording with the RNS implant confirmed high gamma power’s predictive value for symptom severity (87% accuracy) and strong correlation with depression measures, such as the Hamilton Depression Rating Scale (HAMD; r2 = 0.65, P = 1.98 × 10−5) (33). Performing this broader exploration prior to implanting the RNS device enabled contextualization of the biomarker within the patient’s circuit associated with depression.

#### Targeted recording and stimulation

In contrast to exploratory mapping, targeted recording streamlines biomarker detection by focusing on a predetermined structure informed by prior research. For instance, Gill and colleagues capitalized on the role of the amygdala in PTSD in prior electrophysiological studies which demonstrated increased theta band power associated with fear-related memory retrieval in PTSD (34). As such, they implanted RNS electrodes into the amygdala of two individuals with PTSD and found increases in theta power within the amygdala corresponded with a high symptom severity state, corroborating prior research efforts, linking increased theta power to fear-related memory retrieval (39). Likewise, Frank et al. implanted DBS leads into the anterior limb of the internal capsule based on promising findings from prior studies demonstrating the efficacy of DBS therapy in this region for treating OCD and depression. They found an inverse correlation between low and high gamma and depression scores (via VAS-D) (40).

Similarly, for behavior-related disorders, such as OCD and binge eating disorder, studies concentrated on the reward/reinforcement system of the basal ganglia (39, 41). Looking at the ventral capsule/ventral striatum (VC/VS) for OCD, Provenza and colleagues found that delta power was negatively correlated with symptom severity across all three patients whose recordings were analyzed (right VC/VS, r = -0.59, left VC/VS, r = -0.56) (32). In a separate study, Nho and colleagues identified low-frequency signals (<15Hz) on iEEG coinciding with occurrence of obsessive thoughts in both ambulatory and provocatory settings (36). For binge eating disorder, Shivacharan and colleagues found increases in delta within the nucleus accumbens (NAc) corresponded with a high symptom severity state in two patients (35). This finding recapitulated prior work which found an association between anticipation of food reward with the increased low frequency power in the NAc of mice (35, 42).

While the targeted structure approach allows personalized biomarker identification within one structure, preliminary mapping allows the identification of the most relevant biomarker across a patient-specific circuit. Despite these differences, both approaches have been effectively used to guide intracranial stimulation for the treatment of neuropsychiatric disorders.

### Intracranial stimulation as a treatment for neuropsychiatric disorders

Once an electrophysiological biomarker is identified, it is monitored and manipulated using intracranial electrical stimulation with the goal of alleviating the associated neuropsychiatric symptoms. The efficacy of this hinges on factors such as how stimulation is implemented and how the outcomes are evaluated.

#### Stimulation protocol

Stimulation parameters are typically set to provide maximal therapeutic benefit conferred at the lowest possible stimulation settings to avoid patient discomfort and stimulation side effects, as well as to minimize charge and power consumption (43). To modulate the function of identified electrophysiological biomarkers, prior studies have adopted intermittent bursts of high-frequency (>=100 Hz) electrical stimulation, consistent with current clinical practices in setting neuromodulation parameters in DBS for movement disorders and RNS for epilepsy (44, 45). The exact parameters, including amplitude and other settings, are tailored to each patient’s response and tolerability to stimulation. These stimulation protocols are often implemented for an extended period, ranging from 8 weeks to two years. Target engagement, whether or not the stimulus reaches the intended location and sufficiently modulates the region (46), is well characterized in these studies, relative to transcranial approaches, because the same stimulation electrodes are also used for recording. However, retrospective confirmation of the implanted electrode location is not always available, leaving the exact location implanted at the discretion of the treating psychiatrist and neurosurgeon.

#### Outcome evaluation

To evaluate the efficacy of these stimulation protocols, various outcome criteria are compared between the initial weeks of treatment relative to that at the end of the treatment period. This involves clinical assessment, physiological changes, and research-based criteria. For example, Scangos and colleagues assessed outcomes via clinical depression scales, such as HAMD and Montgomery-Asberg Depression Rating Scale (MADRS) (33). For PTSD symptoms, Gil and colleagues tracked clinical assessment scores of the Clinician-Administered PTSD Scale and PTSD Checklist for DSM-5. They found that symptom severity was significantly correlated with amygdala theta band power (34). Provenza and colleagues tracked objective changes in affect through Automated Facial Action Recognition as well as other physiologic measures such as EKG, blood volume pulse, and EEG (32).

#### Overall success rate

The overall success rate was consistently defined by the amount of improvement from pre-treatment assessment to the primary endpoint based on clinical evaluation criteria specific to individual neuropsychiatric conditions (see **Table 4** for more detail). For depression, Scangos and colleagues looked for a change in depression ratings as defined by the MADRS (33). For OCD, research often looks for a change in the Yale-Brown Obsessive-Compulsive Scale (Y-BOSC), for example, whether participants’ scores had shown 35% reduction from baseline in this scale (32). For PTSD, a difference of mean Clinician-Administered PTSD Scale for DSM-5 greater than or equal to 13 is often used as a treatment success criterion (34). For binge eating, Shivacharan and colleagues have defined the primary endpoint as at least 50% of subjects exhibiting a decrease in the number of loss of control eating events per week (35).

Several of these studies also included remission criteria. For example, Scangos and colleagues found their pilot participant met depression remission criteria of MADRS score <10 (33). Provenza and colleagues found 40% of participants (n=5) met the criteria for remission of OCD with a Y-BOCS score of less than or equal to 12 (32). Shivacharan and colleagues found 50% of participants (n=2) met criteria for remission of binge eating disorder with fewer than average of four binge eating events per month over the prior three consecutive months (35). While promising, these results need to be followed up with additional studies to see the larger effect size.

## Discussion

In this review, we examined 5 pioneering studies that integrate intracranial recording and closed-loop stimulation for treating neuropsychiatric conditions. Overall, biomarker identification and stimulation methodologies represented a consistent paradigm across studies. However, our findings indicate a paucity of research focusing specifically on closed-loop approaches. Despite three authors involved in paper selection - one performing the search, two selecting the articles, and one confirming the relevance - potential misses remain possible. The small number of studies limits our ability to make definitive conclusions about the broader applicability and efficacy of these interventions across diverse populations and conditions. Regardless of lingering uncertainties, the potential treatment benefits observed in these 11 participants across these 5 studies underscores the urgent need for further elucidating the mechanisms, treatment efficacy, and long-term viability of this treatment approach.

First, of primary interest, further research should seek to understand the effects of intracranial stimulation in the context of the neuropsychiatric circuitry. Despite numerous past studies utilizing functional neuroimaging, the direct link between brain structures and neuropsychiatric symptoms has remained limited (47, 48). Consequently, many neuropsychiatric symptoms have been reconceptualized as dysfunctions within specific functional networks (49). As such, research that employs preliminary mapping of biomarkers and potential therapeutic sites across broader circuits may enhance neural circuit modulation efficacy. For instance, Scangos and colleagues implanted depth iEEG electrode probes throughout various sites within the emotion circuitry of their patient. Among the electrode locations, they identified amygdala activity as the strongest predictor of depression symptoms. They then performed stimulus-response mapping and discovered that stimulation of the VC/VS led to consistent and sustained symptomatic improvement. To support these findings, they performed evoked potential mapping and found that the VC/VS and amygdala constitute a structurally and functionally connected subnetwork, with the VC/VS influencing numerous distant brain regions (33). This suggests the importance of the VC/VS in modulating the associated network at large, given its significant connections and effectiveness as the optimal site for stimulation (33). This network approach suggests that the success of neuromodulatory treatments hinges on their effects on the entire network (50). Therefore, exploring the broader network as a stimulation target based on preliminary mapping may be crucial for future successes in utilizing intracranial stimulation as a treatment for neuropsychiatric diseases.

Second, future research may also consider complementary approaches to interrogate the heterogeneous neuropsychiatric circuits for effective neuromodulation. Neuropsychiatric symptoms are frequently comorbid across various diseases and widely vary. As such, the Research Domain Criteria (RDoC) was developed as a framework for researchers to explore neuropsychiatric diseases based on broad neurobehavioral functioning domains, rather than operationally defined based on symptoms (51). One key aspect is the characterization of neural circuits that drive specific functional problems (48). In addition to identifying electrophysiological features time-locked to a state of high symptom severity, future research may benefit from probing explicit behavioral tasks that arise from a given functional circuit (1, 52, 53). For instance, dysphoria can be interrogated by electrophysiological responses to emotional imagery tasks, while response inhibition - variably impacted across neuropsychiatric disorders including depression, anxiety, and PTSD - could be examined using the Go/NoGo task (52, 54). In the context of neuromodulation, this approach offers a way to precisely characterize, target, and modulate these behavioral circuits directly. Additionally, modulating the behaviors that arise from these neural circuits may address symptoms common to multiple diseases (55). Bolstering the RDoC framework for closed-loop neuromodulation may provide additional insights into behavior across various disorders, contributing to a better understanding of trans-diagnostic behavioral circuits.

Third, evaluating the long-term viability of this intracranial approach as a treatment for neuropsychiatric conditions is crucial. While some intracranial electroencephalography (iEEG) implants remain in the patient’s brain for an extended period (e.g., RNS), the efficacy of long-term stimulation effects over years remains unknown. For instance, while Scangos and colleagues demonstrated that intracranial effects can reduce patients’ depression symptoms for up to 60 days (33), data from further longitudinal follow-ups are not yet available. It remains uncertain whether direct brain stimulation over an extended period (e.g., a few years) can lead to alterations in the underlying neural circuitry, some of which may even involve structural changes. This raises technical and ethical considerations regarding the extent of this treatment as a long-term solution without introducing potential side effects. Longitudinal follow-up of patients from clinical trials may shed more light on these issues in the upcoming years.

Furthermore, it’s important to note these additional caveats of the intracranial approach. First, the intracranial approach is inherently invasive, significantly limiting its accessibility. Currently, this technique is reserved for individuals with clear treatment resistance; neuromodulation is not intended as a replacement for standard pharmacotherapy but rather as an option for those who haven’t benefited from initial treatments. Given the involvement of pathological networks in symptom production, it may be possible to engage deeper network connections indirectly by modulating cortical projections to influence symptoms effectively. Identifying specific cortical entry points for modulating deeper structures through non-invasive stimulation techniques could represent a promising path for enhancing the applicability and accessibility of this approach in future research. Second, further research is needed to elucidate biomarkers across various patient populations (40). While precision medicine operates at an individual level, identifying common patterns may be necessary to objectively confirm biomarker significance as disease process hallmarks. This will be crucial for scalability across diagnoses and generalizability across demographic groups. Last, identifying the best stimulation targets within the network is essential, considering the possible distinction between the biomarker representing symptomatology and the area offering the best treatment response. Investigating efficient treatment parameters capable of adequately perturbing the targeted circuit is crucial. Collectively, clarifying the mechanisms, treatment efficacy, and long-term viability of the intracranial approach along with addressing these additional caveats will provide necessary insights into neuropsychiatric disorder physiology and contribute to much-needed innovations in future neuropsychiatric treatments.
