# Supplementary material for: Intracranial closed-loop neuromodulation as an intervention for neuropsychiatric disorders: an overview
Source: Front Psychiatry. 2025 Jan 30;16:1479240. doi: 10.3389/fpsyt.2025.1479240 (PMC11821593; doi:10.3389/fpsyt.2025.1479240)
Supplement: Supplementary file 1 [file Table1.pdf]

Supplemental Table 1. Search terms for each database. After PubMed search, MEDLINE was omitted in subsequent searches since its results were already included in PubMed.

Database	Search term
PubMed	("closed-loop neuromodulation"[Title/Abstract] OR "adaptive neuromodulation"[Title/Abstract] OR "closed-loop DBS"[Title/Abstract] OR "adaptive DBS"[Title/Abstract] OR "responsive neurostimulation"[Title/Abstract] OR "intracranial recording"[Title/Abstract] OR "intracranial stimulation"[Title/Abstract]) AND ("Psychiatric Disorders"[MeSH Terms] OR "Mental Disorders"[MeSH Terms] OR "Depression"[MeSH Terms] OR "Obsessive-Compulsive Disorder"[MeSH Terms] OR "PTSD"[MeSH Terms] OR "Anxiety Disorders"[MeSH Terms] OR "Bipolar Disorder"[MeSH Terms] OR "Schizophrenia"[MeSH Terms] OR "psychiatric"[Title/Abstract] OR "mental health"[Title/Abstract]) AND English[lang]
Web of Science	TS=(("closed loop neuromodulation*" OR "adaptive neuromodulation*" OR "adaptive DBS*" OR "closed loop DBS*" OR "intracranial recording*" OR "intracranial stimulation*") AND ("neuropsychiatric disease*" OR "neuropsychiatric illness*" OR "neuropsychiatric disorder*" OR "psychiatric disease*" OR "psychiatric illness*" OR "psychiatric disorder*" OR "mental disease*" OR "mental illness*" OR "mental disorder*" OR depression* OR depressive* OR "Obsessive-Compulsive Disorder*" OR OCD OR "Post-Traumatic Stress*" OR "Post-Traumatic Neuroses" OR PTSD OR anxiet* OR psychotic* OR psychosis OR psychoses OR Schizophren* OR bipolar* OR manic))<br><br>NOT abstract, english, NOT medline, NOT data set
Scopus	TITLE-ABS-KEY (("closed loop neuromodulation*" OR "adaptive neuromodulation*" OR "adaptive DBS*" OR "closed loop DBS*" OR "intracranial recording*" OR "intracranial stimulation*")) AND TITLE-ABS-KEY ("neuropsychiatric disease*" OR "neuropsychiatric illness*" OR "neuropsychiatric disorder*" OR "psychiatric disease*" OR "psychiatric illness*" OR "psychiatric disorder*" OR "mental disease*" OR "mental illness*" OR "mental disorder*" OR depression* OR depressive* OR "Obsessive-Compulsive Disorder*" OR ocd OR "Post-Traumatic Stress*" OR "Post-Traumatic Neuroses" OR ptsd OR anxiet* OR psychotic* OR psychosis OR psychoses OR schizophren* OR bipolar* OR manic*) AND (LIMIT-TO (LANGUAGE , "English")) AND (LIMIT-TO (EXACTKEYWORD , "Humans"))
Embase	((('neuromodulation'/exp OR closed) AND loop AND neuromodulation*:ti,ab,kw OR 'adaptive neuromodulation*':ti,ab,kw OR 'adaptive dbs*':ti,ab,kw OR 'closed loop dbs*':ti,ab,kw OR 'intracranial recording*':ti,ab,kw OR 'intracranial stimulation*':ti,ab,kw) AND ('mental disease'/exp OR 'depression'/exp OR 'obsessive compulsive disorder'/exp OR 'posttraumatic stress disorder'/exp OR 'anxiety'/exp OR 'psychosis'/exp OR 'schizophrenia'/exp OR 'bipolar disorder'/exp OR 'neuropsychiatric disease*':ti,ab,kw OR 'neuropsychiatric illness*':ti,ab,kw OR 'neuropsychiatric disorder*':ti,ab,kw OR 'psychiatric disease*':ti,ab,kw OR 'psychiatric illness*':ti,ab,kw OR 'psychiatric disorder*':ti,ab,kw OR 'mental disease*':ti,ab,kw OR 'mental illness*':ti,ab,kw OR 'mental disorder*':ti,ab,kw OR depression*:ti,ab,kw OR depressive*:ti,ab,kw OR 'obsessive-compulsive disorder*':ti,ab,kw OR ocd:ti,ab,kw OR 'post-traumatic stress*':ti,ab,kw OR 'post-traumatic neuroses':ti,ab,kw OR ptsd:ti,ab,kw OR anxiet*:ti,ab,kw OR psychotic*:ti,ab,kw OR psychosis:ti,ab,kw OR psychoses:ti,ab,kw OR schizophren*:ti,ab,kw OR bipolar*:ti,ab,kw OR manic:ti,ab,kw) AND 'intracranial recording':ti,ab,kw OR 'intracranial stimulation':ti,ab,kw) AND [english]/lim AND ([embase]/lim NOT ([embase]/lim AND [medline]/lim) OR ([embase classic]/lim NOT ([embase classic]/lim AND [medline]/lim))) AND ('article'/it OR 'editorial'/it OR 'review'/it)
PsycInfo/PsycNet	Any Field: (closed loop neuromodulation* OR adaptive neuromodulation* OR adaptive DBS* OR closed loop DBS* OR intracranial recording* OR intracranial stimulation*) AND<br>Any Field: ("neuropsychiatric disease*" OR "neuropsychiatric illness*" OR "neuropsychiatric disorder*" OR "psychiatric disease*" OR "psychiatric illness*" OR "psychiatric disorder*" OR "mental disease*" OR "mental illness*" OR "mental disorder*" OR depression* OR depressive* OR "Obsessive-Compulsive Disorder*" OR OCD OR "Post-Traumatic Stress*" OR "Post-Traumatic Neuroses" OR PTSD OR anxiet* OR psychotic* OR psychosis OR psychoses OR Schizophren* OR bipolar* OR manic) AND<br>Language: English
